# Emotional suppression and depressive symptoms in women newly diagnosed with early breast cancer

**DOI:** 10.1186/s12905-015-0254-6

**Published:** 2015-10-24

**Authors:** Lingyan Li, Yanjie Yang, Jincai He, Jinyao Yi, Yuping Wang, Jinqiang Zhang, Xiongzhao Zhu

**Affiliations:** 1 Medical Psychological Institute, Second Xiangya Hospital, Central South University, Changsha, Hunan 410011 P. R. China; 2 Department of Medical Psychology, Public Health Institute of Harbin Medical University, Harbin, China; 3 The First Affiliated Hospital, Wenzhou Medical College, Wenzhou, China; 4 National Technology Institute of Psychiatry, Central South University, Changsha, China

**Keywords:** Emotional suppression, Depressive symptoms, Breast cancer, Oncology, Women’s health

## Abstract

**Background:**

Patients with breast cancer usually present varying levels of depressive symptoms. Emotional suppression, as a coping style, refers to an individual’s ability to consciously control expression of negative emotions. Thus, emotional suppression is an important psychological factor related to depressive symptoms in patients with breast cancer. It has long been considered that compared to European and American women, Chinese women are more likely to ascribe to norms of negative emotion control for smooth social interaction. However, there is paucity of research focusing on emotional suppression among Chinese women with breast cancer. Thus the aims of the current study were (1) to investigate the incidence of depressive symptoms in women newly diagnosed with early breast cancer in Mainland China, and (2) to examine the relationships between emotional suppression and depressive symptoms in these patients.

**Methods:**

The Center for Epidemiological Studies Depression Scale (CES-D), the Beck Anxiety Inventory (BAI) and the Chinese version of the Courtauld Emotional Control Scale (CECS) were used to assess the level of depressive symptoms, anxiety symptoms and emotional suppression respectively in 247 women with early breast cancer and 362 healthy women. Analyses of variance were conducted to investigate group differences on depressive symptoms and emotional suppression. Bivariate correlations and Hierarchical regression analyses were performed to examine the effect of emotional suppression on depressive symptoms in participants after controlling the impact of group membership and anxiety level.

**Results:**

(1) The incidence rates of clinical and severe depressive symptoms in patients were 36.4 and 36.0 % respectively. (2) Patients scored significantly higher than healthy women on CECS. (3) The scores on CECS were significantly associated with the total CES-D scores in all participants; Anger suppression significantly predicted the total CES-D scores.

**Conclusions:**

The majority of women newly diagnosed with early breast cancer reported clinical or severe depressive symptoms. As well, these patients presented a controlled emotion coping style. Emotional suppression was associated with the level of depressive symptoms in women newly diagnosed with breast cancer. Anger suppression might play a unique role in the depressive symptoms among women newly diagnosed with breast cancer.

## Background

Breast cancer has been the most common malignancy amongst women around the world [1]. In 2008, about 1.38 million women were diagnosed with breast cancer and approximately 0.46 million women died of the disease worldwide [2]. China used to be a low-incidence area of breast cancer, however, urban cancer registries have documented 20 to 30 % increases in the incident rates of breast cancer in the last decade [3]. Based on data of the sixth national population census and the recent national incidence of female breast cancer, it is estimated that more than 270,000 Chinese women face the risk of being diagnosed with breast cancer.

Being diagnosed with breast cancer is a catastrophic life event, which usually induces psychological distress. Extensive studies have shown that patients with breast cancer usually present varying levels of depressive symptoms [4, 5]. For example, 5 to 15 % of post-surgery patients report depressive symptoms using structured interviews. These percentages rise to 15 to 30 % when using screening questionnaires [6]. Even years after diagnosis and treatment, some patients with breast cancer still suffer from significant clinical depression [7, 8]. In addition to decreasing physiological and psychological function [9–12], depressive symptoms may have other serious consequences, such as increasing mortality in patients with breast cancer [13].

Various factors are related to depressive symptoms in patients with breast cancer, including demographics, disease-related factors, and individual psychosocial characteristics such as coping styles and personality traits [14–17]. Emotional suppression, one of these coping styles, is defined as an individual’s ability to consciously control the expression of negative emotions, such as anxiety, sadness, and anger. It is a core component of the cancer personality trait, commonly referred to as type C behavioral pattern elaborated by Temoshok [18]. Empirical evidence has been accumulated to support the link between emotional suppression and psychosocial maladjustment such as depressive symptoms in patients with breast cancer [19]. For example, Schlatter et al. [20] found that anger suppression was associated with higher level of depression in breast cancer patients during chemotherapy. Classen et al. [21] found that emotional suppression as a whole was related to worse mood in women with advanced breast cancer. Conversely, attempts to express negative emotions could lead to better adjustment to cancer [18, 22]. Emotional expression in a supportive group environment enhanced the management of disease-related emotion and reduced distress. These findings suggest that a controlled emotion coping style may hinder psychological adjustment to cancer. In other words, sufficient emotional expression is important for breast cancer patients.

However, research to date has focused on western women. Studies show that compared to European and American women, Chinese women are more likely to ascribe to norms of emotional control for ensuring smooth social interactions [23]. Thus, emotional control appears to have important implication for the psychological adjustment of Chinese patients with breast cancer. Despite its importance in the Chinese context, there was only one study conducted by Ho and colleagues [24] concerning Hong Kong Chinese women. Ho et al. reported that women with cancer showed a tendency to suppress emotion, and emotional suppression positively predicted stress level even after the effect of depressed mood was under control. But almost ten years has passed since then, no evidence has indicated the relationships between emotional suppression and depressive symptoms in women newly diagnosed with breast cancer in Mainland China. Figuring out the role of emotional suppression may help us to better understand the depressive symptoms in patients, and to adopt useful intervention strategies to improve their psychological adjustment. Thus the aims of the current study were: (1) to investigate the incidence of depressive symptoms in the women newly diagnosed with early breast cancer in mainland China, and (2) to examine the relationships between emotional suppression and depressive symptoms in these patients.

## Methods

### Sample and design

#### Patient group

Between September 2011 and June 2012, patients were recruited from Xiangya Hospital and Second Xiangya Hospital, Central South University. Eligible patients met the following criteria: (1) women newly diagnosed with breast cancer stage I or stage II by biopsy, (2) informed of the diagnosis by their relatives or doctors, and (3) ability to speak Chinese. Patients with the following conditions were excluded: (1) breast cancer recurrence, (2) known untreated or unstable major medical conditions other than breast cancer, (3) known major psychiatric or neurological disorders that would interfere with completion of the measures, and (4) history of substance abuse.

Two hundred and fifty-five women met the first three inclusion criteria and were invited to the study. Eight of them were excluded from the study according to the exclusion criteria. Thus, the final patient sample consisted of 247 women, all of whom agreed to participate after being informed of the aim and procedure of the study. Participants aged from 26 to 70 years, with a mean (SD) = 47.45(7.43) years. About 40.5 % of the patients were from urban areas and 59.5 % were from rural areas. Most (89.5 %) of the patients were married, 7.3 % were divorced, and 3.2 % were widowed. They had received a mean (SD) of 9.77(3.42) years of schooling. The employment status of the patients reported were: employed (77.3 %), housewife (15.4 %), and retired (7.3 %).

#### Healthy comparison group

We recruited the healthy women from Changsha and the surrounding area for comparison. Eligible women had self-reported good physical health and spoke Chinese. Women with the following conditions were excluded: (1) history of any type of cancer, (2) known untreated or unstable major medical conditions, (3) known major psychiatric or neurological disorders that would interfere with completion of the measures, and (4) history of substance abuse.

Three hundred and eighty-one women volunteered to participate. Nineteen of them were excluded from the study, leaving 362 women as health controls. The ages of participants ranged from 25 to 68 years, with a mean (SD) = 46.59 (7.57) years. Among the 362 women, 39.2 % were from urban areas and 60.8 % were from rural areas. The majority (93.1 %) of the healthy women were married, 4.4 % were divorced, and 2.5 % were widowed. They had received a mean (SD) of 9.53 (3.43) years of schooling. The employment status reported were: employed (74.3 %), housewife (10.8 %), and retired (14.9 %).

### Procedure

The research procedure of the current study was approved by the Ethics Committee of the Second Xiangya Hospital, Central South University. After participants provided written informed consent, trained psychology students administered structured questionnaires in face-to-face interviews to collect information on demographic, emotional control, anxiety and depressive symptoms.

### Measures

#### Demographics form

Demographic data including: age, years of schooling, long-term residence, marital status and employment status.

#### Center for Epidemiological Studies Depression Scale (CES-D)

The CES-D is a 20-item self-report questionnaire that assesses depressive symptoms in general population. For each item, individuals are asked to describe how often they have experienced a given symptom over the last week, and respond on a 4-point scale ranging from 0 (not at all) to 3 (a lot). Higher scores indicate higher levels of such symptoms. The CES-D possesses strong reliability and validity in clinical and nonclinical adults [25, 26]. The cronbach’s α coefficient of CES-D in current study was 0.88.

#### The beck anxiety inventory (BAI)

The BAI is a 21-item self-report inventory that measures the severity of anxiety symptoms in the last week. Items are rated on a 4-point scale ranging from 0 (not at all) to 3 (severely, I could barely stand it), with higher scores indicating higher levels of anxiety symptoms [27]. Good psychometric properties have been demonstrated in a variety of clinical populations, including breast cancer patients [28]. The cronbach’s α coefficient of BAI in current study was 0.87.

#### The Chinese version of courtauld emotional control scale (CECS)

The original CECS was developed to assess the extent of emotional control when a particular negative emotion is experienced. It contains 21 items separated into three subscales for the report of the suppression or the expression of feelings towards anger, anxiety, and unhappiness (depressed mood). Individuals are asked to response to the phrases such as “When I feel angry, I bottle it up” on a 4-point scale ranging from 1 (almost never) to 4 (almost always). The total emotional suppression scores are summed by three subscale scores, with higher scores indicating higher levels of emotional suppression. In previous research, the CECS has shown good reliability [29].

The Chinese version of CECS was developed through the procedures of translation and back translation, as well as reference to the version developed by Ho et al. [24] to ensure the meaning of each statement corresponded accurately to the meaning of the original one. In our previous research, the Chinese version of CECS was proved to be applicable for Chinese women with breast cancer [30]. In the current sample, CECS and its subscales exhibited strong internal consistency: 0.96 (CECS), 0.89 (anger), 0.91 (anxiety), and 0.92 (depressed mood).

### Analyses

Descriptive analyses, analysis of variance, and hierarchical regression analyses were performed by SPSS 17.0 software with a 5 % level of significance. The incidence rates of depressive symptoms were calculated according to the cutoff CES-D score of 16 established by Radolff: the CES-D score of 16 to 26 indicating clinical depressive symptoms, and CES-D score ≥27 indicating severe depressive symptoms [31]. Analyses of variance were conducted to investigate differences on depressive symptoms and emotional suppression tendencies between two groups. Bivariate correlations between age, years of schooling, emotional suppression, anxiety symptoms and depressive symptoms for all participants were analyzed using Pearson correlations or Spearman correlations depending on the distribution of variables. Hierarchical regression analyses were performed to examine the effect of the three emotional suppression tendencies on depressive symptoms in participants. As anxiety may be closely related to depressive symptoms, we controlled levels of anxiety in our regression analyses. Collinearity between independent variables was tested based on variance inflation factors and tolerances [32].

## Results

### Demographic data

Table 1 showed demographic characteristics of each group. There was no statistically significant difference between patients and healthy women in demographic variables except for the employment status.

**Table 1:** Demographic data of the two groups

	Patient group	Healthy comparison group	_F or_ *χ* ^2^	*p*
	(*n* = 247)	(*n* = 362)		
Age (years)	47.45 ± 7.43	46.59 ± 7.57	1.911	0.167
Years of schooling	9.77 ± 3.42	9.53 ± 3.43	0.713	0.399
Educational level (%)			1.021	0.796
Primary school	19.8	20.2		
Junior high school	47.0	43.6		
Senior middle school	17.0	19.9		
College and above	16.2	16.3		
Residence (%)			0.097	0.755
Urban	40.5	39.2		
Rural	59.5	60.8		
Marital status (%)			2.670	0.263
Married	89.5	93.1		
Widowed	3.2	2.5		
Divorced	7.3	4.4		
Employment status (%)			9.875	0.007
Employed	77.3	74.3		
Housewife	15.4	10.8		
Retired	7.3	14.9		

### Descriptive statistics

Table 2 showed the descriptive statistics for depression symptoms, anxiety symptoms and emotional suppression. The mean (SD) score of CES-D in patients was 21.58 (9.82). More than one third (36.4 %) of the patients had scores of 16 or higher, and 36.0 % reported scores of 27 or higher. The mean (SD) score of CES-D in healthy women was 14.90 (6.65). About 34.8 % of the healthy women had scores of 16 or higher, and only 5.2 % of them had scores of 27 or higher. The total CES-D scores were significantly different between two groups (*p* < 0.001; Cohen’s d = 0.80). The prevalence of three levels of depressive symptoms were significantly different between two groups (*χ*^2^ = 111.530; *p* < 0.001); patients had lower incidence rate of no depressive symptoms and higher rate of severe depressive symptoms.

**Table 2:** CES-D, BAI and CECS means and standard deviations

	Patient group	Healthy comparison group	F	Cohen’s d
	(*n* = 247)	(*n* = 362)		
Depression symptoms	21.58 ± 9.82	14.90 ± 6.65	100.303***	0.80
Anxiety symptoms	13.58 ± 7.15	6.93 ± 7.36	122.854***	0.92
Anger Suppression	19.03 ± 5.87	15.26 ± 3.73	93.855***	0.77
Anxiety Suppression	18.56 ± 5.94	16.05 ± 3.67	41.674***	0.51
Depression Suppression	18.63 ± 6.06	15.79 ± 4.06	48.149***	0.55
Total emotional suppression	56.23 ± 17.12	47.09 ± 9.93	69.066***	0.65

***p < 0.001

The total BAI scores were significantly different between two groups (*p* < 0.001; Cohen’s d = 0.92). Significant differences on three emotional suppression tendencies and total emotional suppression were found between patients and healthy women (*p* < 0.001; Cohen’s d ranged from 0.51 to 0.77).

### Bivariate correlations between variables

We examined the relationships between age, years of schooling, emotional suppression, anxiety symptoms and depressive symptoms, and Pearson correlation coefficients and Spearman correlation coefficient were presented in Table 3. Age was not significantly correlated with other variables (*p* > 0.05). Years of schooling was significantly negatively correlated with emotional suppression tendencies, and Spearman correlation coefficients ranged from −0.10 to −0.18 (*p* < 0.05). Pearson correlation coefficients between the three subscales of CECS ranged from 0.55 to 0.64 (*p* < 0.01).

**Table 3:** Bivariate correlation coefficients between variables

	Depressive symptoms	1	2	3	4	5	6
1. Age	0.01	—					
2. Years of schooling	−0.05	−0.02	—				
3. Anxiety Symptoms	0.75**	0.05	−0.06	—			
4. Anger Suppression	0.66**	−0.01	−0.10*	0.54**	—		
5. Anxiety Suppression	0.50**	−0.02	−0.18**	0.39**	0.55**	—	
6. Depression Suppression	0.58**	−0.02	−0.16**	0.45**	0.64**	0.60**	—
7. Total emotional suppression	0.62**	−0.03	−0.16**	0.50**	0.83**	0.81**	0.85**

*p < 0.05, **p < 0.01

Emotional suppression tendencies were found to be significantly positively correlated with depressive symptoms and anxiety symptoms. Pearson correlation coefficients between CECS subscales and depressive symptoms ranged from 0.50 to 0.66 (*p* < 0.01) while Pearson correlation coefficients between CECS subscales and anxiety symptoms ranged from 0.39 to 0.54 (*p* < 0.01). Anxiety symptoms were significantly positively correlated with depressive symptoms (*p* < 0.01).

### Hierarchical regression analyses

In order to further explore the effect of the three emotional suppression tendencies on depressive symptoms in women with breast cancer, multiple regression analyses were performed based on the correlation; the results were shown in Table 4. The regression equation for model 1 was significant when group membership (1 = patients, 2 = controls) was the sole predictor of depressive symptoms (*F* = 100.303, *p* < 0.001). The regression equation for model 2 was also significant when anxiety symptoms were entered (*F* = 404.997, *p* < 0.001). The final model was significant when emotional suppression tendencies were entered (*F* = 230.751, *p* < 0.001), with a R^2^ change of 0.085, indicating that emotional suppression accounted for 8.5 % of the variance of depressive symptoms after controlling the effects of group membership and levels of anxiety symptoms. However, the regression coefficient was not significant for depression suppression and anxiety suppression, whereas anger suppression had significant effect on depressive symptoms among breast cancer patients (*p* < 0.05).

**Table 4:** Regression analysis for emotional suppression in predicting depressive symptoms

	B	SEB	*t*	F	R^2^	Adjusted
						R^2^
Step 1				100.303***	0.142	0.140
Group	−6.682	−0.377	−10.015***			
Step 2				404.997***	0.572	0.571
Anxiety symptoms	0.787	0.719	24.682***			
Step 3				230.751***	0.657	0.654
Anger suppression	0.464	0.269	5.473***			
Depression suppression	0.159	0.094	1.977			
Anxiety suppression	−0.006	−0.003	−0.083			

***p < 0.001

## Discussion

Previous studies have found that most breast cancer patients report psychological distress and may be in need of emotional help [33], especially during the first 24 months after diagnosis [34]. In current study, the incidence rates of clinical depressive symptoms and severe depressive symptoms in women newly diagnosed with breast cancer were 36.4 and 36.0 % respectively. These rates are higher than findings from previous studies using the same instrument for assessing the depressive symptoms in patients under later-stages treatment for breast cancer [15, 35]. Our findings suggest that patients who are newly diagnosed with breast cancer are at higher risk of developing depressive symptoms. Nevertheless, methodologically more rigorous assessments (e.g., clinical interview) should be conducted to determine if these patients meet the criteria for clinically significant depression.

Some researchers have argued that emotional suppression may be an important risk factor for cancer. Individuals who adopt a style of emotion suppression in response to negative emotions throughout adult life are more likely to be diagnosed with breast cancer [36]. Similar findings have been found in other diseases [37]. The current study showed that women with breast cancer in Mainland China used higher level of emotional suppression than healthy women. Our findings suggest that a relative high level of emotional suppression is not conducive to women’s health, even though forbearance, the mastering of maintaining harmonious relations with others, is generally considered a valuable character trait in women according to traditional Chinese culture [23]. Supportive–expressive therapy for those diagnosed with cancer in previous study may also be helpful for breast cancer prevention in mainland China, and future research should focus on this field.

Consistent with previous research [21], examination of the correlations between the emotional suppression and depressive symptoms revealed that participants with controlled emotional coping style have higher level of depressive symptoms. Findings from regression analyses implicated emotional suppression had a significant effect on the depressive symptoms; higher scores on anger suppression predicted higher level of depressive symptoms after controlling for the effects of group membership and levels of anxiety symptoms. Depression suppression and anxiety suppression had no significant effect on depressive symptoms, though both subscale scores were significantly correlated with CES-D scores. These results, which were similar to findings of Iwamitsu’s study [38], implied that suppression of different negative emotion had different psychological outcomes. Therefore, it is important to encourage suppressive patients to express negative emotion correctly and appropriately.

Anger suppression has been found to be linked with increased risk of adverse cardiac events, greater pain, and poor immune function [39]. In current study, when considering the three emotional suppression tendencies concurrently, anger suppression showed a unique role in predicting depressive symptoms in Chinese women with breast cancer. However, Ando et al. [40] found that only anxiety suppression could significantly predict the level of psychological stress of breast cancer patients before the diagnosis. The possible reason for this inconsistency may be that anger expression is more socially undesirable for Chinese women and they seem to have more difficulty in expressing anger than anxiety and depression. As mentioned by Ho et al., Hong Kong Chinese cancer survivors had higher levels of anger suppression compared to British counterparts [24]. What is more, patients are considered to be angry when realizing the authenticity and immutability of a diagnosis of life-threatening disease. As a result, psychosocial intervention in breast cancer patients targeting anger expression may be effective for alleviating symptoms of depression. Further studies are required to confirm the effect of anger expression on psychological distress.

### Strength and limitation

A key strength of the present study was that we recruited a matched control group for comparison, a methodological improvement from previous studies. High rates of participation (98.9 and 95.0 % of patients and controls, respectively) and complete response on all of the questionnaires significantly helped to reduce errors caused by missing data. Finally, our study was the first to illustrate the importance of assessing emotional suppression tendencies in Chinese women newly diagnosed with breast cancer.

There were several limitations worth noting of the current study. Firstly, because of the self-report nature of the CES-D, it was difficult to draw conclusions about clinically diagnosed depression, which should be included in the future study. Secondly, measures of depressive symptoms were only taken at one time point. Future studies with multiple time points would help to explore the relationships between emotional suppression tendencies and changes in depressive symptoms over time in breast cancer patients.

## Conclusions

The findings of this study suggested that the majority of Chinese women newly diagnosed with early breast cancer had clinical or severe depressive symptoms, and that these patients presented a controlled emotion coping style. Our study lends further empirical evidence that emotional suppression is associated with the level of depressive symptoms in women newly diagnosed with breast cancer. Finally, anger suppression may play a unique role in predicting symptoms of depression in women with breast cancer.
